# Easy Removal of Epiphytic Bacteria on *Ulva* (Ulvophyceae, Chlorophyta) by Vortex with Silica Sands

**DOI:** 10.3390/microorganisms10020476

**Published:** 2022-02-21

**Authors:** Xiaojie Liu, Jin Zhao, Peng Jiang

**Affiliations:** 1 CAS and Shandong Province Key Laboratory of Experimental Marine Biology, Center for Ocean Mega-Science, Institute of Oceanology, Chinese Academy of Sciences, Qingdao 266071, China; liuxiaojieyx@163.com (X.L.); zhaojin@qdio.ac.cn (J.Z.); 2 Laboratory for Marine Biology and Biotechnology, Qingdao National Laboratory for Marine Science and Technology, Qingdao 266237, China; 3 College of Earth Sciences, University of Chinese Academy of Sciences, Beijing 100049, China

**Keywords:** associated bacteria, seaweed, silica sand, surface sterilization, *Ulva*

## Abstract

Macroalgae-associated bacteria play an important role in their algal hosts’ biological processes. They are localized on surfaces of the host thalli, as well as between and even within algal cells. To examine the differences in community structures and functions between epi- and endo- bacteria, an effective approach for maximizing epiphyte removal from delicate seaweeds while retaining endophyte fidelity must be developed. In this study, a variety of surface sterilization methods for *Ulva prolifera* were compared, including mechanical, chemical, and enzymatical treatments. According to the results of scanning electron microscope (SEM) and denaturing gradient gel electrophoresis (DGGE) analysis, almost complete removal of epiphytic bacteria on *Ulva* was obtained simply by co-vortex of seaweeds with silica sands, causing minimal disturbance to endosymbionts when compared to previous published methods. In addition, the adaptability was also confirmed in additional *U. prolifera* strains and *Ulva* species with blade-like or narrow tubular thallus shapes. This easy mechanical method would enable the analysis of community composition and host specificity for *Ulva*-associated epi- and endo-bacteria separately.

## 1. Introduction

Macroalgal-bacterial associations were demonstrated to be widely distributed in marine habitats [1]. Bacterial communities have been shown to play a key role in terms of algal growth [2], nutrition acquisition [3], resistance to biofouling [4], adaptation to environmental stresses [5], spore germination, and colonization of seaweeds [6], and may even act as an indispensable factor in algal morphogenesis process [7,8,9,10,11,12]. Macroalgae-associated bacteria were found on surfaces of host thalli, as well as between or even within algal cells [13,14]. These two groups of bacteria were known as epiphytes and endophytes, respectively. However, the differences in bacterial composition and host specificity between these two groups of bacteria have not been of concern until recently [15,16,17,18]. To quantitatively compare this variation using high-throughput sequencing techniques, it was essential to develop an effective method that can remove epiphytic bacteria as much as possible while simultaneously maintaining the fidelity of the endosymbiotic bacteria.

Among reported enzymatic and chemical methods applied to macroalgae, the UNSET buffer (urea and SDS as principal ingredients) and 3M^TM^ Rapid Multi-Enzyme Cleaner (Sydney, NSW, Australia) were designed for selective collection of epiphytic bacteria [19,20]. Although both of them were successful in obtaining representative samples for epiphytes, the staining with fluorescent dye 4′,6-diamidino-2-phenylindole (DAPI) still showed that some bacteria remained on the outer surface of algae, which made the algal thalli not suitable for the extraction of endophytes [21]. To make the endophytes completely free from epiphytic contamination, the bleach-ethanol treatment was employed to obtain epiphytes-free macroalgae [22,23]. However, this protocol was too aggressive for delicate algae and would greatly reduce the abundance of endophytes [21]. A more balanced protocol was developed in which the macroalgae were treated with cetyltrimethylammonium bromide (CTAB) and proteinase K at 60 °C for 30 min, followed by incubation with Umonium Master (bactericidal cleanser) overnight [21]. However, high-temperature treatment could alter the abundance of endophytes communities to some extent [24] and was likely to significantly affect further transcriptome analysis. Thus, for delicate seaweeds, an effective method to meet the demand to maximize the removal of epiphytes, while causing little disturbance to endophytes, is still missing.

Species of green seaweeds *Ulva* (Ulvophyceae, Chlorophyta) often have the simple shape of either blade-like or tubular thalli, and they are typical opportunistic seaweeds. Once the environmental conditions are suitable, they can grow rapidly, sometimes causing massive green tides and serious economic losses [25,26]. In addition, some morphological variations which may benefit their floating lifestyle, were always observed during the blooming stage [27,28,29]. It has been hypothesized that the associated bacteria with *Ulva* spp. may contribute greatly to the blooming of their hosts, possibly by providing assistance on nutrition acquisition [17,30] or inducing floating-adaptive morphology. In this study, we attempted to develop an efficient protocol to maximize the removal of the epiphytic bacteria while maintaining the native endophytic community for the blooming-causative green seaweed *U. prolifera* O.F. Müller [31,32], facilitating the analysis of community composition and host specificity for *Ulva*-associated epi- and endo-bacteria separately.

## 2. Materials and Methods

### 2.1. Algal Samples

Two floating *U. prolifera* strains, S1 and S3, were collected from the Qingdao coast during the blooming periods of green tides in 2007 and 2014, respectively, and an attached strain S2 was collected from Lianyungang coast in 2011. Unialgal cultures of S1 and S2 were grown in Von Stosch’s Enriched Medium at 20 °C under a 12:12 h light:dark cycle with a photon flux rate of 60 μmol m^−2^ s^−1^. Strain S3 was frozen at −20 °C right after sampling. The attached specimens of *U. linza* Linnaeus and *U. compressa* Linnaeus were sampled from the Qingdao coast in 2014 just before the sterilization experiments.

### 2.2. Sterilization and SEM Detection

As shown in Table 1, cultured samples from strain S1 of *U. prolifera* were subjected to a single or a combination of several mechanical, enzymatic or chemical sterilization protocols. Silica sands (Ruijinte Ltd. Com., Beijing, China) in two grain sizes, 60–125 µm and 125–250 µm, were obtained by grinding and gradient screening. To evaluate the adaptability of the optimized method for other *Ulva* spp., we applied it to two other strains of *U. prolifera*, i.e., S2 and S3 (normal and frozen samples respectively) as well as two other species including *U. linza* and *U. compressa*. Each protocol was followed by three times of brief wash and vortex step in sterile seawater. All treatments were repeated in triplicate, and effects on both epiphytes removal and intactness of algal cell wall were assessed using the scanning electron microscope (SEM, S-3400N, Hitachi, Tokyo, Japan), which was a more stringent method than DAPI staining. SEM detection was performed following the protocols described by Callow (1978) [33]. All tissues were fixed by 5% glutaraldehyde for 12 h, then gradient dehydration was carried out with 30, 50, 80, 90, and 100% ethanol at room temperature for 15 min. Samples were then dried at a critical point, coated with gold-palladium by direct-current sputtering, and examined under SEM.

### 2.3. Denaturing Gradient Gel Electrophoresis

Based on the results of SEM, some groups were chosen for further denaturing gradient gel electrophoresis (DGGE) analysis. The sterilized thalli in each selected group containing DNA from all endophytes and any potentially remained epiphytes were grounded in liquid nitrogen prior to a total DNA extraction following a CTAB protocol [34]. In addition, the DNA templates for epiphytes solely was extracted from the seawater remained after the sterilization process [19]. PCR was conducted employing the universal bacterial primers F338 (5′-CCTACGGGAGGCAGCAG-3′) and R518 (5′-ATTACCGCGGCTGCTGG-3′) to amplify the V3 region of 16S rRNA gene [35]. A GC-clamp was coupled to the forward primer to improve DGGE separation. Amplifications were performed in volumes of 50 µL containing 10 × PCR buffer 5 μL, dNTP (2.5 mM) 3.2 μL, rTaq 2 units, GC-338F (20 μM) 1 μL, 518R (20 μM) 1 μL, template DNA 50 ng. After an initial denaturing step at 94 °C for 5 min, 30 cycles of denaturing (94 °C, 1 min), annealing (55 °C, 45 s) and extension (72 °C, 1 min) were completed, followed by a final amplification step at 72 °C for 10 min. Successful amplification of the V3 region was verified by the agarose gel electrophoresis. DGGE analysis was conducted using the DCode Universal Mutation Detection System device (Bio-Rad, Hercules, CA, USA). Optimal electrophoretic separation was obtained using 35–55% denaturing gradient polyacrylamide gels, running for 300 min at 150 V in 1 × TAE buffer at a constant temperature of 60 °C. After silver staining, the gels were visualized and digitally captured via the Gel-Doc2000 (Bio-Rad, Hercules, CA, USA). Each selected sample was detected by DGGE in duplicate. The variation of bacterial abundance and diversities among groups were examined based on the DGGE profile.

## 3. Results and Discussions

**Figure 1:**
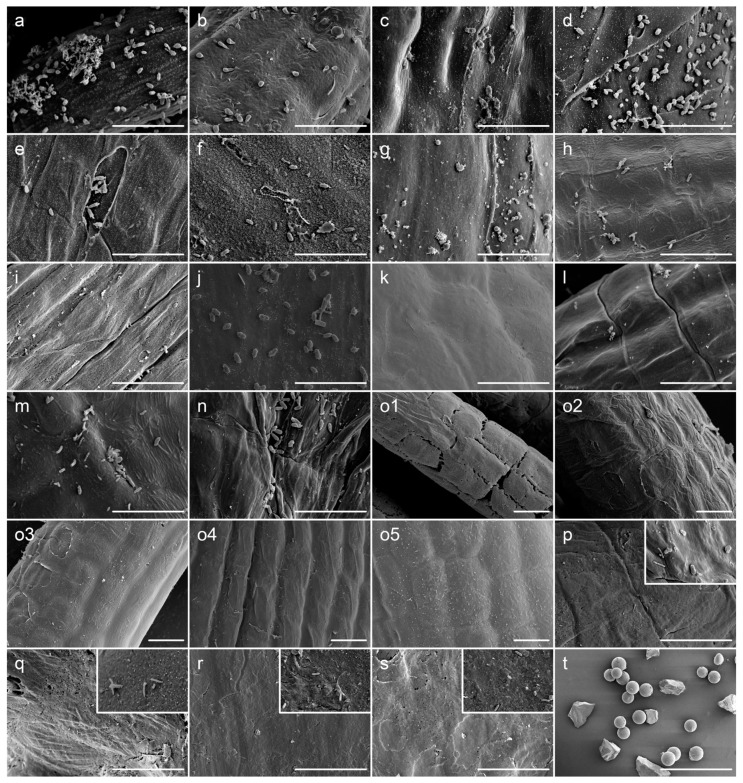
SEM detection for effects of removal of epiphytes by various methods. The characters in each photograph indicate the group described in Table 1 except for group t which was a mixture of glass beads and silica sand. For group (**p**–**s**), the control without sterilization was embedded at the top-right corner using the same scale bar. Scale bars = 10 µm (group (**a**–**s**)), or 1 mm (group (**t**)).

**Figure 2:**
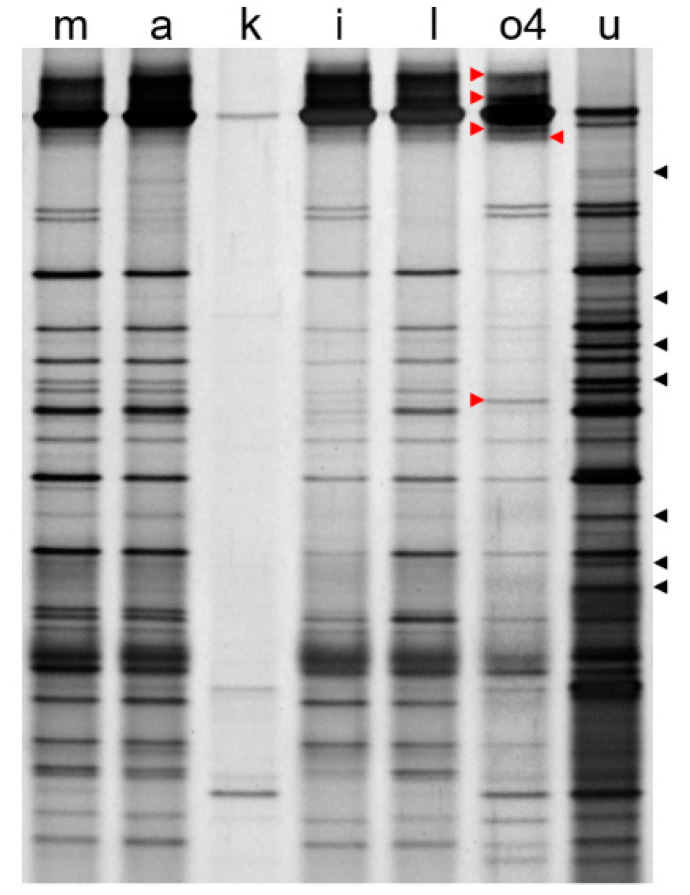
DGGE profiles of 16S rDNA V3 fragments amplified from selected groups. The characters on top of each lane indicated the groups described in Table 1 except for group u, in which the DNA template representing epiphytes was extracted from the remaining seawater left by group o4. Red arrows indicated partial specific bands from potential endo-bacteria which were absent in group u. Black arrows indicated partial specific bands from potential epi-bacteria which were absent in group o4.

The SEM observation showed that attempts to eliminate the epiphytes from *Ulva* spp. by means of lysozyme, papain, snailase, cellulase, lysozyme + papain + snailase, rapid multienzyme cleaner, ethanol, ultrasonic bath sonication, or glass beads-vortex were all unsuccessful (Figure 1c–h,j,m,n). All of these methods were successful in reducing epiphytes to some extent, but a significant fraction remained evident under SEM detection. These results were consistent with those of DGGE by which the observed patterns in these groups were quite similar to the control (lane m, a; Figure 2), indicating the presence of bulk of epiphytes. In general, various enzymatic sterilization protocols were designed to either directly break up the bacterial cell wall, or to degrade the extracellular polymeric substance (EPS) consisting of polysaccharides, carbohydrate, and proteins produced by bacteria [36]. It was suggested that EPS usually formed a network by which the epiphytes may be tightly associated with the host cells [37], making them difficult to be removed completely from the macroalgae [38]. This may explain the failure in most groups using limited mechanical forces or a single type of enzyme.

For proteinase K or UNSET buffer treatment group, it was difficult to detect bacteria-shaped particles under the SEM (Figure 1i,l), implying that high-temperature treatment may contribute greatly to the degradation of EPS and elimination of epiphytes. However, some variations on the abundance of putative endosymbionts were revealed by the results of DGGE (lane i, l; Figure 2). Consequently, if these methods were applied to extract the DNA templates for transcriptome analysis with whole *Ulva* endosymbionts, the risk of distortion resulted from high-temperature treatment could not be ignored. Moreover, due to the obvious seasonal alternation of epiphytic bacterial structures on *Ulva* [15], the corresponding potential variations on the EPS components also might limit the utilization of enzymatic or chemical methods.

Of all of the chemical biocides for biofilm control, chlorine was the most commonly used strong oxidizing agent and disinfectant [39]. Sodium hypochlorite has been shown to be effective for surface sterilization against red, green or brown seaweeds [23], and has been used for endosymbiont composition analysis for green alga *Caulerpa* which has highly differentiated morphology [22]. In this study, the bleach method using sodium hypochlorite can get rid of all epiphytes in seconds (Figure 1k), but it was too aggressive for *U. prolifera* that took the form of monostromatic thallus without a protective surface, causing the endosymbionts to almost completely disappear (lane k; Figure 2). This phenomenon has also been observed in another delicate green alga *Bryopsis* [21].

In contrast to all of the other methods tested, the results showed that a newly developed mechanical method (i.e., co-vortex of *Ulva* thalli with sterilized silica sands at room temperature) was the best method to balance the maximized removal of epiphytes and minimized interference to the endophytes. The results of SEM showed that the cleaned seaweeds with almost complete removal of epiphytes could be obtained by our method using silica sands of 60–125 µm or 125–250 µm diameter. Since silica sands with smaller size were found to have obvious damage to *Ulva* cell walls (Figure 1(o1)), the optimized parameters (i.e., vortex at 3200 rpm for 2 × 15 min with silica sands in size of 125–250 µm) were determined by comparisons. It was indicated that this protocol could keep almost no epiphytes remained on the surface of *Ulva* thalli even for frozen samples (Figure 1(o4,o5)), and the adaptability was verified among other *U. prolifera* strains (Figure 1p,q), as well as other *Ulva* species that were in the shape of either blade-like or tubular thalli (Figure 1r,s). Compared to glass beads in a similar size (Figure 1t), more irregular shapes of sand particles may contribute to the success of this new method by generating greater friction force. More importantly, when comparing the lane o4 and lane u from DGGE gel in Figure 2, some specific bands could be identified in each lane, implying the existence of specific endo- or epi-bacteria. It was believed that the bands shared in these two lanes might come from a very small number of epiphytes remains after vortex, but they were more likely to come from those non-specific strains in terms of spatial localization, which were distributed both on the surface and inside the algal thalli, as happened in the drifting *Sargassum horneri* [18]. In addition, the results of DGGE also clearly showed that the silica-sands group (lane o4; Figure 2) got much richer signals than the bleach group (lane k; Figure 2), indicating that vortex treatment had the least potential interference with endosymbionts, which would be beneficial for the 16S rRNA metabarcoding or transcriptome analysis of this special bacterial group.

It has to be pointed out that the specific parameter of this developed protocol was only suitable for algae with simple shapes such as *Ulva*. For algal species with complex structures, there was no doubt that a certain number of epiphytes were likely to remain after treatments. Therefore, further parameters optimization or careful selection of algal segments would be necessary. In these situations, even if complete removal of epiphytic bacteria could not be achieved, the significant reduction in the abundance of epiphytes was still helpful since it might allow the low abundant endophytic bacterial populations to be detected. Meanwhile, the co-vortex with glass beads has been used to remove the epiphytes in red macroalga *Gracilaria* [40], which indicates that this kind of mechanical method (i.e., co-vortex with particles) is generally applicable across species in seaweeds and can be preferentially employed to analyze the community structure of epiphytes using 16S rRNA metabarcoding.

## 4. Conclusions

In summary, an efficient, rapid, and easy mechanical method to almost completely remove the epiphytic bacteria from fresh or frozen *Ulva*, and to keep the fidelity of endophytes to the greatest extent, was developed. This method was capable of analyzing the community composition and host specificity between these two types of bacterial groups simultaneously or providing epiphytes-free living algae thalli (especially for those species with simple shapes).

## Figures and Tables

**Table 1:** Protocols applied for the surface sterilization of *Ulva* thallus.

Type	Group No.	Sterilization Method	Extended Protocol
Control	a	no	fresh S1, control for groups from c to o4
	b	no	frozen S1, control for group o5
Enzymatic	c	lysozyme (AMRESCO, Solon, OH, USA)	S1 + 2 mg/mL lysozyme in SSW ^1^, 37 °C × 1 h
	d	papain	S1 + 2 mg/mL papain in SSW, 55 °C × 30 min
	e	snailase	S1 + 2 mg/mL snailase in SSW, 37 °C × 1 h
	f	cellulase	S1 + 2 mg/mL cellulase in SSW, 25 °C × 2 h
	g	lysozyme + papain + snailase	S1 + mixture of lysozyme, papain, and snailase (2 mg/mL each) in SSW, 37 °C × 1 h
	h	rapid multienzyme cleaner	S1 + EDTA and filter-sterilized rapid multienzyme cleaner (3M, North Ryde, NSW, Australia) in CFASW ^2^, room temperature × 2 h
	i	proteinase K	S1 + 2 mg/mL proteinase K in SSW, 60 °C × 30 min
Chemical	j	ethanol	S1 + 75% ethanol, room temperature × 5 min
	k	bleach	S1 + 3% sodium hypochlorite, room temperature × 30 s
	l	UNSET buffer	S1 + 1 mL UNSET buffer, 55 °C × 15 min ^3^
Mechanical	m	ultrasonic bath sonication	S1 in ultrasonic cleaner, 40 kHz × 15 min
	n	glass beads (Bio-Rad, Hercules, CA, USA)-vortex	0.03 g S1 + 0.3 g glass beads (120–250 µm) in SSW, vortex in 1.5 mL tube at 3200 rpm, 2 × 20 min
	o1	silica sand-vortex	0.03 g S1 + 0.3 g silica sand (60–125 µm) in SSW, vortex in 1.5 mL tube at 3200 rpm for 2 × 1 h
	o2	silica sand-vortex	same as group o1 except for size of silica sand (125–250 µm)
	o3	silica sand-vortex	same as group o2 except for 2 × 30 min
	o4	silica sand-vortex	same as group o2 except for 2 × 15 min
	o5	silica sand-vortex	same as group o4 except for using frozen S1
	p	silica sand-vortex	same as group o4 except for using frozen S3
	q	silica sand-vortex	same as group o4 except for using S2
	r	silica sand-vortex	same as group o4 except for using *U. compressa*
	s	silica sand-vortex	same as group o4 except for using *U. linza*

^1^ SSW, sterile seawater. ^2^ [19]. ^3^ [20].

## Data Availability

Not applicable.
